# Lorlatinib in the treatment of a rare pulmonary mucoepidermoid carcinoma with EML4-ALK fusion: a case report and literature review

**DOI:** 10.3389/fonc.2024.1415254

**Published:** 2024-08-29

**Authors:** Zijun Xu, Xiaofeng Cong, Ziling Liu

**Affiliations:** Cancer Center, The First Hospital of Jilin University, Changchun, China

**Keywords:** pulmonary mucoepidermoid carcinoma (PMEC), EML4-ALK fusion, lorlatinib, anaplastic lymphoma kinase-tyrosine kinase inhibitor, first line treatment

## Abstract

Pulmonary mucoepidermoid carcinoma (PMEC) is a rare tumor with limited clinical data available due to its low incidence. So far, there are no universal treatment guidelines for this malignant tumor. We present here the case of a 59-year-old female never smoker who was initially referred to our hospital with cough and hemoptysis and was eventually diagnosed with PMEC. Based on further genetic testing, echinoderm microtubule-associated protein-like4-anaplastic lymphoma kinase (EML4-ALK) fusion variants E20:A20 (V2) was found. The patient was treated with lorlatinib as the first-line treatment. This case is the first to describe the effectiveness of lorlatinib in treating an advanced high-grade PMEC with EML4-ALK fusion V2 mutation patient.

## Introduction

Mucoepidermoid carcinoma (MEC) is a malignant tumor composed of epidermoid cells, mucinous cells and intermediate cells in different proportions, with a mixed structure of solid and cystic (1, 2). MEC usually occurs in salivary glands, especially parotid glands, while uncommonly originates in the lung. PMEC accounts for 0.1%-0.2% of primary pulmonary malignant tumors (3), and the first human case was published in 1952. It is often misdiagnosed as adenocarcinoma, squamous cell carcinoma, or adenosquamous carcinoma (4). Due to its low incidence, the clinical characteristics and optimal treatment options of PMEC have not been fully reached a consensus. With the development of immunohistochemistry and genetic analysis technology, the rate of missed diagnosis and misdiagnosis of PMEC has gradually decreased. Meanwhile, the rapid evolution of targeted drugs provides new treatment options for PMEC. This article reports a rare case of advanced high-grade PMEC with EML4-ALK fusion who achieved a remarkable response to third-generation anaplastic lymphoma kinase-tyrosine kinase inhibitors (ALK-TKI) lorlatinib.

## Case report

**Figure 1:**
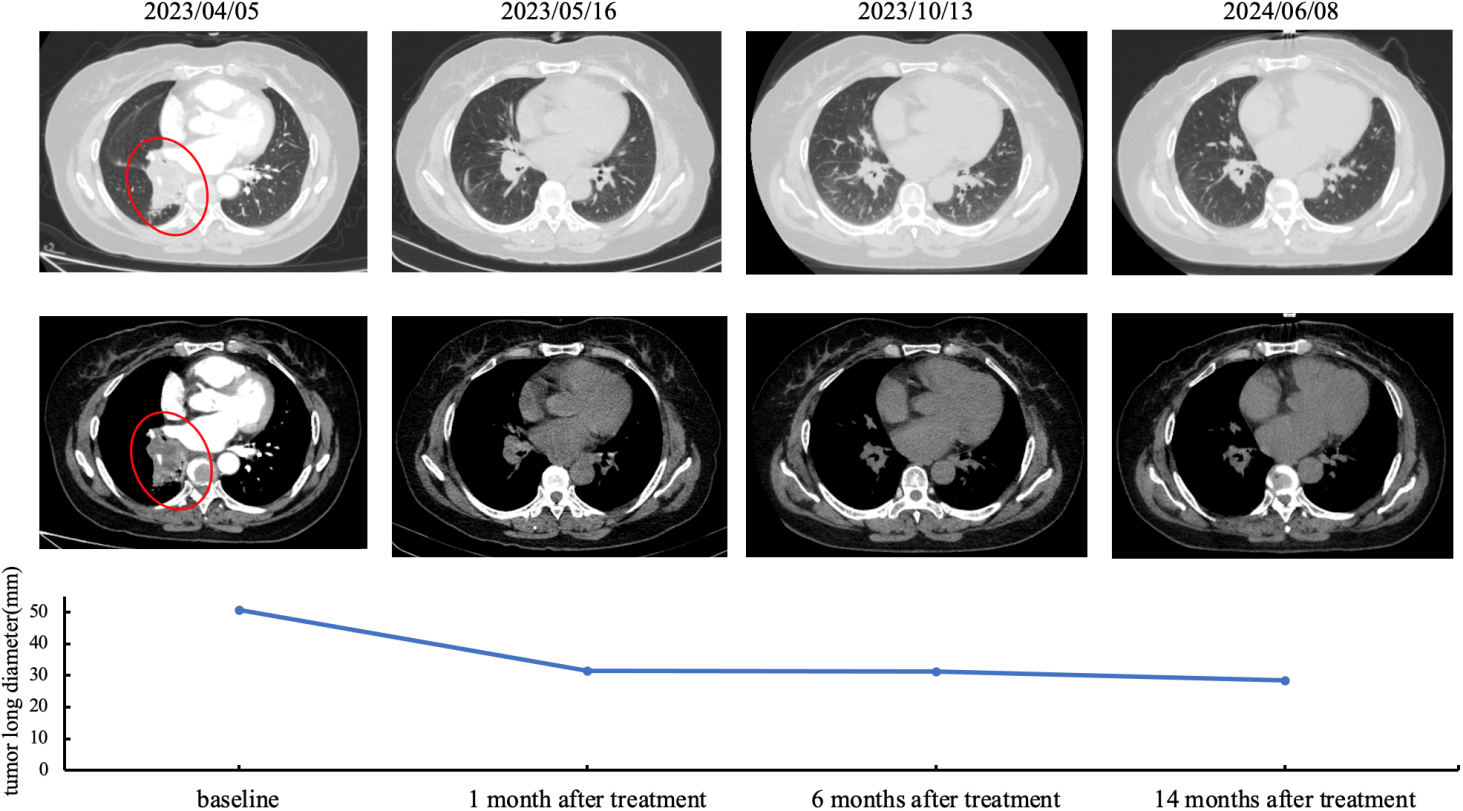
Treatment response was evaluated by chest CT imaging after lorlatinib. Representative CT images obtained with the lung window (upper row) and mediastinal window (lower row). Red circle indicates the location of the tumor on the lower lobe of the right lung. Line chart demonstrate the change of tumor long diameter (mm).

**Figure 2:**
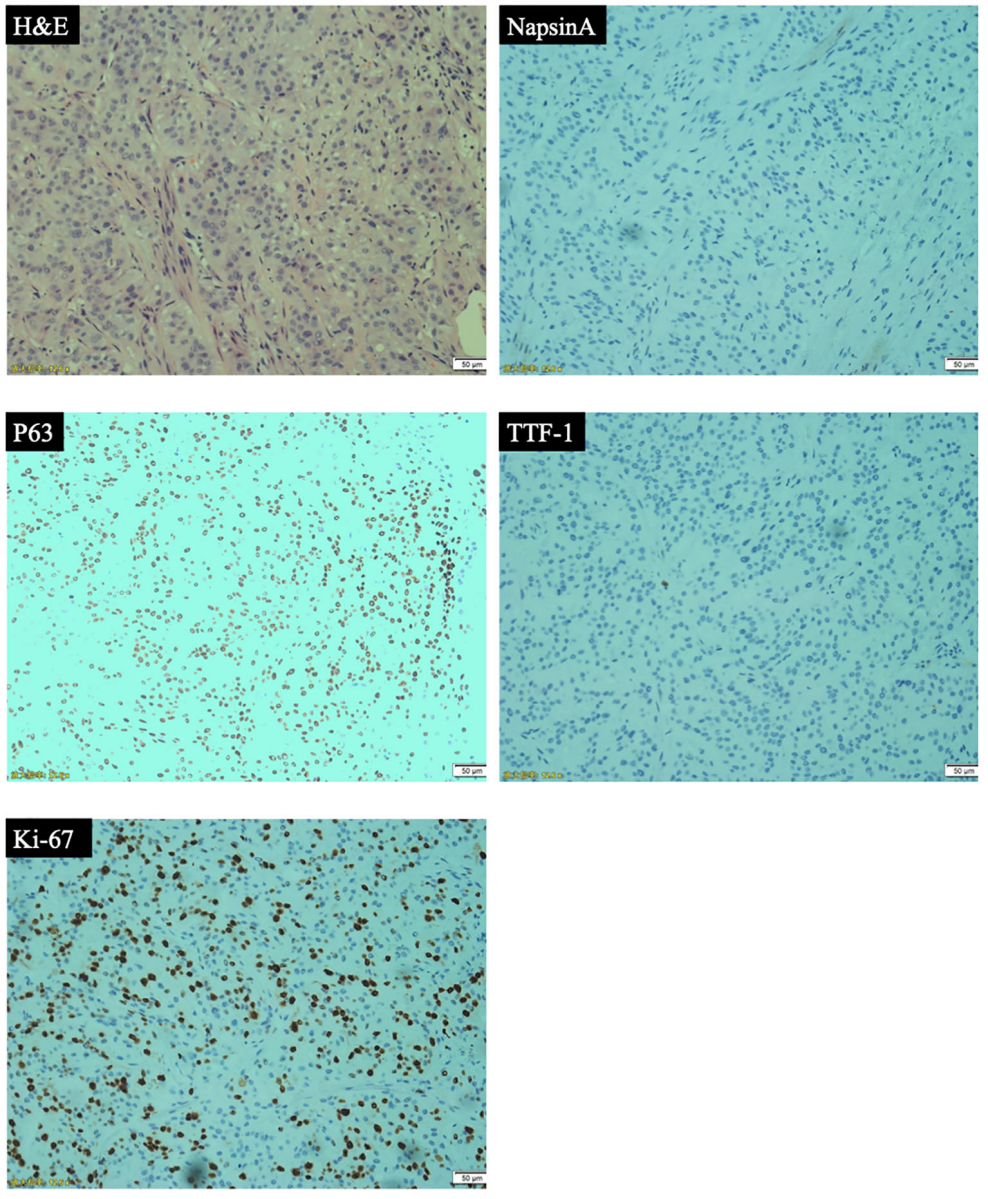
Pathological analyses: hematoxylin and eosin (H&E) staining and immunohistochemical (IHC) analyses (representative IHC results of NapsinA, p63, TTF-1, Ki-67).

Patient was a 59-year-old female who did not smoke and had no surgical history or previous medical history such as cancer history other than lung cancer, and family history of lung cancer. The patient was admitted to our hospital because of repeated cough for 3 months and streaking of sputum with blood discontinuously for 1 month, denying symptoms such as fever, dizziness, vomiting, blood in the stool or breathing difficulties. There were not significant weight loss and loss of appetite. Chest computed tomography (CT) (**Figure 1**) showed the wall of the middle segment bronchus, middle lobe and lower lobe bronchus of the right lung was thickened, with stenosis and local truncation of the lumen. A mass of high-density shadow with the unclear nodule border was found in the lower lobe of the right lung. The size was 3.1cm × 4.2cm, and the CT value was about 28HU. The boundary between the lesion and the adjacent interlobar pleura was not clear, and the lesion wrapped around the arteries and veins of the lower lobe of the right lung, with patchy high-density shadows at the distal end. There were nodular hyperdense shadows observed in the upper lobe of both lungs, the middle lobe of the right lung, and the lower lobe of the left lung, with the size of 0.2-0.9cm. Magnetic resonance imaging (MRI) and CT of the abdomen and pelvis did not reveal a space-occupying lesion. The patient underwent bronchial biopsy and brushing, the histopathological examination revealed non-small cell carcinoma in the basal segment of the right lower lobe. A large number of neutrophils, small amount of glandular epitheliums, squamous epithelium and macrophages were found in the specimens. The results of immunohistochemical (IHC) staining (**Figure 2**) were suggestive of high-grade MEC. IHC analysis showed CK (AE1/AE3) (+), CK7 (-), p63 (+), TTF-1 (-), NapsinA (-), Ki67 (positive rate 40%), GATA3 (+), HER-2 (0), CK20 (-), and ER (-). These results suggested that the pathological stage was T4N2M1a and the clinical stage was IVA.

**Figure 3:**
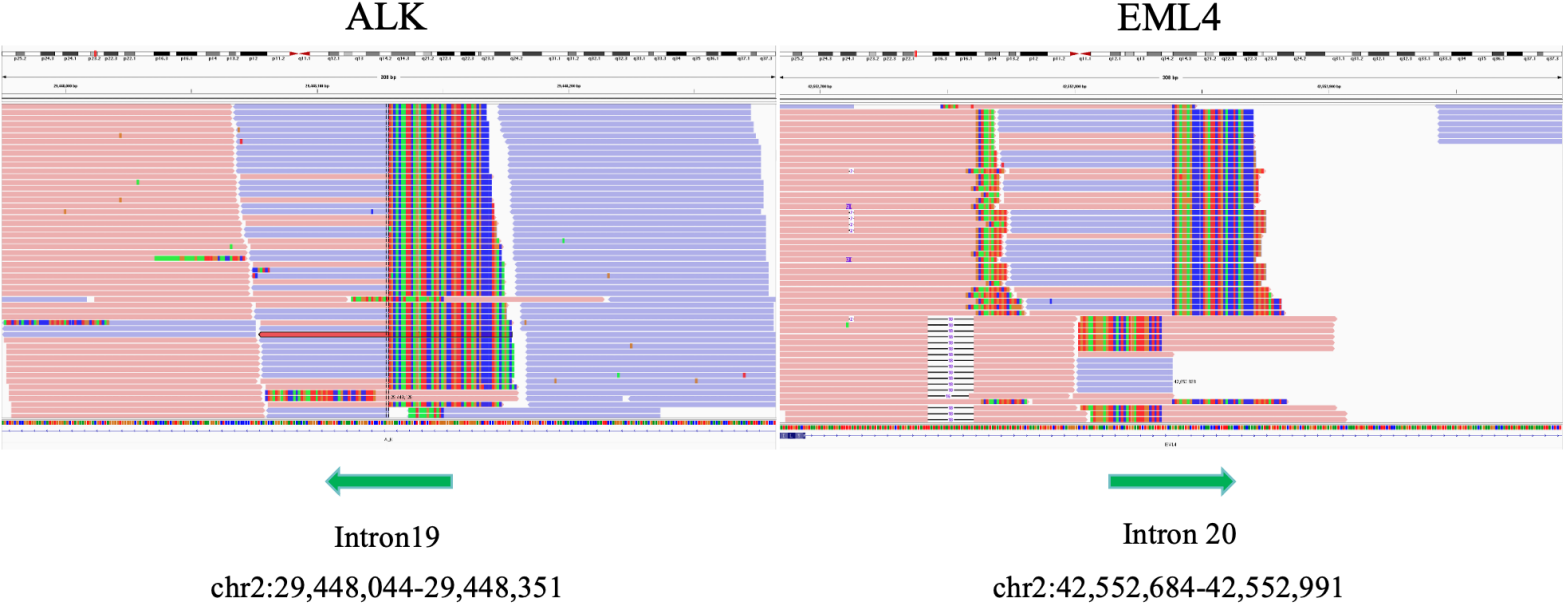
Integrative genome visualization(IGV) of the mutant gene sequence, showing the identification of EML4-ALK.

Further detection of next generation sequencing (NGS) revealed EML4-ALK fusion variants E20:A20 (V2) (**Figure 3**). No mutations were detected in genes that are significantly mutated in lung cancer, such as BRAF, EGFR, ERBB2, KRAS, MET, NTRK1, NTRK2, NTRK3, RET and ROS1. The captured samples were sequenced on MGISEQ-2000 using a panel consisting of 769 genes. The diagnosis and prognosis were explained, and the patient received targeted therapy. The patient was treated with lorlatinib 100mg once daily orally. The response was evaluated by chest CT imaging at 1, 6 and 14 months after lorlatinib, showing that the tumor volume was reduced (**Figure 1**). At the indicated time points, the tumor long diameters were 50.79mm, 31.58mm, 31.18mm and 28.51mm, respectively. Importantly, we achieved a prominent partial response (PR) as the tumor volume decreased more than 37% after 1 month treatment and the trend of tumor shrinkage was observed over the entire 14-month evaluation period. It was worth noting that the reduction of tumor growth became more apparent as the tumor surrounded the trachea actually. There was a noticeable improvement in the patient’s symptoms with no adverse reactions. Therefore, the patient continued the current regimen and followed up regularly.

## Discussion

We report a case of advanced PMEC, a female patient with high-grade tumor tissue differentiation and EML4-ALK fusion variants E20:A20 (V2), who was in treating with lorlatinib as the first line therapy and observed progression-free survival (PFS) over 14 months as of June 8, 2024.

PMEC is a rare malignant tumor of the lung. Previous reports have suggested that the majority of PMEC patients are male, and there is no significant correlation between the incidence of PMEC and smoking (5). According to the World Health Organization (WHO)2021 histological classification of lung cancer, it is a subtype of salivary gland-type lung cancer. Regarding the differentiation grade (6), the low-grade tumors are mostly cystic components without obvious cell atypia, most tumors had no necrosis area, and calcification could be seen microscopically. The central mass or obvious enhancement is common in imaging. Histologically, the high-grade tumors are more likely to have atypical cells, mitotic phase, necrotic areas, and regional lymph node metastasis. The imaging manifestations of high-grade tumors are mostly peripheral masses with unclear boundaries and low enhancement. IHC staining shows negative results for TTF-1 and Napsin A and the positive expression of CK-7, P63, P40 and AB/PAS. Furthermore, Ki-67 ranges from 2% to 80%, it can be used as auxiliary diagnostic indicators for the differentiation of high and low-grade PMEC. Specifically, the higher the index is, the higher the grade level is (7, 8). HER-2 also tends to be highly expressed in high-grade tumors, which may guide future application of targeted anti-HER-2 therapy (9). The most important prognostic factor of PMEC is histological grade, followed by pathological stage and clinical stage. Low-grade PMEC rarely metastasize, with a 5-year disease-specific survival (DSS) rate of 95%. High-grade PMEC have a metastasis rate of 55-80%, with a 5-year DSS rate of 65% (10). Lymph node metastasis is more common in male patients or high-grade tumors (11), and it mainly occurs in organs such as lung, bone and brain.

Due to the limited clinical data of PMEC, there is no relevant treatment and management guidelines. For early solitary lesions, surgery is the main treatment option (12), and for high-grade PMEC, postoperative therapy may be required. At present, the application of chemotherapy and radiotherapy is still controversial (13).

In recent studies (14), researchers have explored the therapeutic targets, and about 40% of MEC patients have EGFR mutations and can benefit from EGFR-TKI. Interestingly, gefitinib may also effective in patients without EGFR mutations. Further studies (15) found that about 65% of PMEC had a novel fusion oncogene, CRTC1-MAML2, combines exon 1 of CRTC1 on chromosome 19p13 with exons 2-5 of the MAML2 gene on chromosome 11q21, which can interrupt normal cell growth and differentiation and up-regulate EGFR ligand dual regulatory protein. Gefitinib has shown dual regulatory protein dependent activity in non-small cell lung cancer (NSCLC) cell lines. Targeting this fusion oncogene has achieved a good clinical response in the treatment of chronic myeloid leukemia (CML), however its application in PMEC needs further research and exploration.

ALK gene mutation is rare in MEC. Compared with the precision diagnosis of EGFR mutations, ALK mutations have not yet fully moved towards the path of precision treatment. In the process of ALK gene fusion (16), the breakpoint of ALK gene is relatively conservative (exon 20), but it has many fusion partners. The most common molecular chaperone in NSCLC is EML4 gene (about 95%). There are different fusion variants according to EML4 gene subtype, and the most common fusion variants are variant 1 (V1, (E13; A20) followed by variant 3 (V3a/b, (E6a/b; A20), and variant V2 has a very low incidence.

Lorlatinib (17), a third-generation ALK-TKIs, is an adenosine triphosphate (ATP)-competitive inhibitor with an optimized structure compared with second-generation drugs. With the combination of small molecule and macrocyclic amide, the structure is more compact, the buried surface area is increased, and it can almost completely enter the center of the ligand pocket of the ATP binding site. The interaction with the kinase domain is stronger and the binding is more stable, and the occurrence of drug resistance is reduced. At the same time, the drug has smaller molecular weight and improved lipophilicity, which is beneficial to reduce the efflux of P-glycoprotein (P-gp) and enhance the ability to cross the blood-brain barrier. According to these characteristics, lorlatinib received accelerated approval in November 2018 for the second- or third-line treatment of ALK-positive metastatic NSCLC. On March 3, 2021, the Food and Drug Administration (FDA) granted regular approval to lorlatinib for patients with metastatic NSCLC whose tumors are ALK-positive, detected by an FDA-approved test (18). National Comprehensive Cancer Network (NCCN) guidelines include loratinib as first-line treatment for patients with advanced or metastatic ALK-rearranged NSCLC. Recently, 2024 American Society of Clinical Oncology (ASCO) reported the long-term follow-up result of phase 3, randomized, open-label CROWN study achieving an amazing over 60 months median PFS, which aimed to evaluate lorlatinib in treatment-naïve patients with advanced ALK-positive NSCLC (19). Based on above guidelines and studies, we decided to use loratinib and looked forward to evaluating the longer-term efficacy.

## Conclusion

We report a case of a female patient with advanced PMEC, accompanied by EML4-ALK fusion, who was treated with loratinib. To the best of our knowledge, this is the first time that a third-generation ALK-TKI has been used in PMEC patients, which provides new treatment options for lorlatinib in the clinical application of PMEC. This underscores the need for further genetic testing in rare types of lung cancer and individualized treatment strategies to avoid missed opportunities for early clinical diagnosis and treatment, especially when the specificity of morphological or immunophenotypic analysis is poor.

## Data Availability

The original contributions presented in the study are included in the article/supplementary material, Further inquiries can be directed to the corresponding author/s.
